# Assessment of Maternal Satisfaction and Associated Factors among Parturients Who Underwent Cesarean Delivery under Spinal Anesthesia at University of Gondar Comprehensive Specialized Hospital, Northwest Ethiopia, 2019

**DOI:** 10.1155/2020/8697651

**Published:** 2020-10-12

**Authors:** Samuel Debas Bayable, Seid Adem Ahmed, Girmay Fitiwi Lema, Debas Yaregal Melesse

**Affiliations:** ^1^Department of Anesthesia, College of Medicine and Health Sciences, Debre Berhan University, Debre Berhan, Ethiopia; ^2^Department of Anesthesia, College of Medicine and Health Sciences, University of Gondar, Gondar, Ethiopia

## Abstract

**Background:**

Spinal anesthesia is the most common anesthetic technique for cesarean delivery. Patient satisfaction is a subjective and complicated concept, involving physical, emotional, psychological, social, and cultural factors. Regular evaluation of maternal satisfaction related to anesthesia service is an important parameter to the required changes and expansion of high-quality care services. We aimed to assess maternal satisfaction and associated factors among parturients who underwent cesarean delivery under spinal anesthesia.

**Methods:**

Institutional-based cross-sectional study was conducted from February to May 2019. A total of 383 parturients were enrolled to assess maternal satisfaction using a 5-point Likert scale. Both bivariable and multivariable logistic regression analyses were done. Variables of *p* value ≤0.2 in the bivariable analysis were a candidate for multivariable logistic regression. A *p* value <0.05 was considered as significantly associated with maternal satisfaction at 95% CI.

**Results:**

This study revealed that 315 (82.3%) of the parturients were satisfied. Single spinal prick attempts (AOR = 2.08, 95% CI = 1.05–4.11), successful spinal block (AOR = 7.17, 95% CI = 3.33–15.43), less incidence of postdural puncture headache (AOR = 2.36, 95% CI = 1.33–4.20), and prophylactic antiemetic use (AOR = 0.35, 95% CI = 0.19–0.66) were positively associated with maternal satisfaction.

**Conclusions:**

The overall maternal satisfaction receiving spinal anesthesia was considerably low. Single spinal prink attempts, successful spinal block, and less incidence of postural puncture headache can increase maternal satisfaction. Therefore, effective perioperative management, skillful techniques, and using the small-gauge Quincke spinal needle (25–27 gauge) may increase the maternal satisfaction and quality of spinal anesthesia management.

## 1. Background

Spinal anesthesia is a safe anesthetic technique for cesarean delivery which gained worldwide acceptance since the introduction to clinical practice [1, 2]. It has good quality of analgesia and avoids general anesthesia-related maternal morbidity and mortality, decreased risk of gastric aspiration, avoids exposure of anesthetic depressant drugs to the neonate, less incidence of deep venous thrombosis, and decreases blood loss during surgery [2–4]. In addition, it has favorable effects on bonding the mother to the newborn [5].

Rate of cesarean delivery has been raising all over the world with a rate of 13–39% [6]. The overall institutional rate of the national population-based cesarean delivery in Ethiopia was 15–18%, which reaches 46% in the private sectors, among which maternal indications accounted for 66%, and others were fetal indications [7].

During preoperative anesthetic evaluation, it is important to explain the procedure, side effects, and possible complications of SA to parturients and obtain informed consent, and this process may improve retention of information and increase maternal satisfaction [8–10]. In addition, emotional support before spinal anesthesia for cesarean delivery helps to decrease preoperative anxiety and increase maternal satisfaction [11, 12].

A study done in South Africa concludes that integrating preanesthesia explanations, counseling during labor, and the use of adequate medications to reduce discomfort, pain, and shivering may increase maternal satisfaction with spinal anesthesia for CD [13].

Studies done in Australia and Kenya on obstetrics surgeries revealed that parturients being illiterate, multiparous, and absence of comorbidities significantly increase maternal satisfaction [14, 15]. In addition, a similar study conducted in Canada showed that being young age, good coordination, and absence of complications were the most influential factors in determining parturient satisfaction [16].

Previous studies regarding maternal satisfaction after SA for cesarean delivery showed that paraesthesia, multiple prick attempts, needle prick pain, intraoperative hypotension, failed block, use of prophylactic antiemetic, intraoperative vomiting, inadequate analgesia, and headache were major obstacles for client satisfaction after spinal anesthesia [2, 4, 5, 10, 17, 18].

Although spinal anesthesia provides excellent anesthesia and analgesia that improve patients' satisfaction, fewer patients (18–20%) still experience some degree of pain and discomfort during the procedure [10], with possible cause of differences in patients' perception of pain, previous experience, race, ethnicity, and experience of the anesthetist [19].

Nowadays, determining the level of client's satisfaction and identifying factors that can affect the level of satisfaction on the health system are the part and parcel of every institution. In University of Gondar Comprehensive Specialized Hospital, a large number of cesarean deliveries are performed every year based on the maternal and fetal indication; however, there was no study related to the level of maternal satisfaction regarding to anesthesia service. Therefore, this institutional-based cross-sectional study was designed to assess the level of maternal satisfaction and identify factors that can affect the maternal satisfaction after receiving spinal anesthesia for cesarean delivery.

## 2. Materials and Methods

### 2.1. Study Design and Setting

After obtaining the ethical approval from the University of Gondar, College of Medicine and Health Sciences, School of Medicine, Ethical Review Committee with reference number SOM/140/02/2019, an institutional-based cross-sectional study was conducted from February to May 2019 among parturients who underwent CD under spinal anesthesia in University of Gondar Comprehensive Specialized Hospital. Nowadays, the hospital holds 550 beds, of which 58 beds are served for obstetric admission. Currently, there are two functional operation rooms for only cesarean delivery.

### 2.2. Study Participants and Data Collection Procedure

After obtaining written informed consent, all parturients who underwent CD under spinal anesthesia during the study period were enrolled in the study. Parturients who had communication problems due to different reasons including neurologic, psychiatric ill parturients, multiple pregnancies, parturients with functional incapacitated systemic illness, headache prior to cesarean delivery, or conversion from spinal to general anesthesia during surgery were excluded from the study.

Rapid preoperative assessment and explanation regarding the techniques of anesthesia, possible side effects, and its management were provided by the assigned anesthetist to the parturient in emergency cesarean delivery. However, in elective cesarean delivery, detailed preoperative assessment and explanations about the techniques of anesthesia, possible side effects, and its management were provided. Besides, question and answer regarding all aspects of anesthesia-related service from the patient was also entertained since the anesthetist and the parturients had adequate time to address and cover all concerns.

In emergency cesarean delivery, intravenous catheter was inserted before arrival at the operating theater, and 10 ml/kg of crystalloids was preloaded. Patients were then placed in the sitting position at the operating table, and a standard integrated monitor was attached. Spinal anesthesia was performed by using a 21–25 G Quincke needle via the L3-4 or L4-5 interspace following 1% lidocaine infiltration. Anesthesia was provided with 10–12.5 mg isobaric bupivacaine of 0.5% with ±10 *μ*g intrathecal fentanyl. Intraoperative management, such as management of nausea and vomiting, heat loss, fluid status, and blood pressure, was at the discretion of each anesthetist.

Data were collected by two junior anesthetists who had not been responsible to manage parturients during the study period. Data were collected through chart review, direct observation, and pretested semistructure questionnaires. Data were collected at two phases: during intraoperative period and 24 hours after delivery. The semistructured questionnaire was developed based on different studies. The questionnaire included two sections: the first section focused on sociodemographic variables, maternal and newborn-related factors, and intraoperative anesthesia- related factors, and the second section contained two parts; postoperative anesthesia-related factors, patient experience, and items of patients' satisfaction were assessed by using a 5-point Likert scale. The five-point Likert scale was adapted from the Leiden perioperative care patient satisfaction questionnaire (LPPSq) and dichotomized as satisfied and dissatisfied based on the demarcation threshold formula. This scale was applied for sub and overall maternal satisfaction, so based on the formula, patients who scored less than 54 points out of 90 were considered as not satisfied whereas 54 and above were considered as satisfied. Leiden perioperative care patient satisfaction questionnaire (LPPSq) was a valid and reliable assessment tool to assess perioperative patient satisfaction [16].

### 2.3. Study Variables

#### 2.3.1. Dependent Variable

Levels of maternal satisfaction through a 5-point Likert scale (1 = completely dissatisfied, 2 = dissatisfied, 3 = neutral, 4 = satisfied, and 5 = completely satisfied) were considered as the dependent variable.

#### 2.3.2. Independent Variables

Maternal and newborn-related factors such as ASA status, history of comorbidity, anesthesia history, parity, pregnancy, indication, urgency of surgery, sex of the newborn, weight of the newborn, APGAR score, status of the newborn, and gestational age were considered as the independent variables. Intraoperative anesthesia-related factors included spinal prick attempt, block height ≤T4, needle prick pain, paraesthesia, surgical pain, surgical duration, difficult to breathe, hypotension, light headedness, bradycardia, intraoperative nausea/vomiting, shivering, failed block, antiemetics, and sedatives or analgesics. Postoperative anesthesia-related factors consisting of PDPH, shivering, PONV, lower back pain, pain with 2 hours immediately after operation were independent variables of this study.

### 2.4. Operational Definitions

  Maternal satisfaction: parturients were considered to be satisfied who scored greater than or equal to the cut-point based on the demarcation threshold formula [20–22]:

(1) $$\begin{array}{c} \text{demarcation threshold formula}=\frac{\left(\text{total highest score}-\text{total lowest score}\right)}{2}+\left(\text{total lowest score}\right). \end{array}$$

  A failed block: the need to repeat spinal anesthesia or intravenous analgesic drug was required to proceed with the surgical procedure.
  Needle prick pain: it is defined as sudden and sharp pain accompanying needle puncture. Paraesthesia is defined as uncomfortable pain accompanying needle.
  Hypotension: a decrease in mean arterial pressure 20% from baseline, and bradycardia is defined as a decrease in heart rate 20% from baseline.
  Postdural puncture headache (PDPH): frontal and/or occipital headache that appears after lumbar puncture, which worsens within 15 minutes of assuming the upright position and improves within 30 minutes of resuming the recumbent position.

### 2.5. Sample Size, Sampling Technique, and Data Analysis

The sample size was determined using the single population proportion formula:

(2) $$\begin{array}{c} \;n=\frac{{\left(z\alpha /2\right)}^{2}pq}{\varepsilon^{2}}, \end{array}$$

where *n* = the desired sample size, *Z* = 1.96 (corresponds to the 95% confidence level), *p* = population proportion (50%, 0.5), and *q* which is 1 − *p*, 1–0.5 = 0.5. *ε* = degree of accuracy (marginal error is 5% (0.05)); then, the sample size is

(3) $$\begin{array}{c} \;n=\frac{{\left(1.96\right)}^{2}\times 0.5\left(1-0.5\right)}{{\left(0.05\right)}^{2}}=384.16∼385. \end{array}$$

Data were checked for completeness, inconsistencies, and then coded and entered using EPI data version 4.4. Then, the data were cleaned and analyzed using SPSS version 23. Descriptive statistics were computed to determine frequencies and summary statistics (mean, standard deviation, median, IQR, and percentage). Data were presented using tables and graphs. All variables with *p* ≤ 0.2 in the bivariable logistic regression analysis were included in the final model of multivariable logistic regression analysis in order to control all possible confounders. Multicollinearity was checked to see the linear correlation among the independent variables by using the standard error. Variables with a standard error >0.2 were dropped from the multivariable logistic regression analysis. Model fitness was checked with the Hosmer–Lemeshow test. Adjusted odds ratio with 95% CI was estimated to identify the factors associated with adherence status using multivariable logistic regression analysis. Level of statistical significance was declared at *p* value <0.05.

## 3. Results

### 3.1. Sociodemographic Characteristics

A total of 383 parturients were enrolled in this study. The mean age and standard deviation of parturients was 27.7 ± 4.9 years. The median and interquartile range of BMI of parturients was 25 (23–27 kg/m^2^) (Table 1).

### 3.2. Maternal and Newborn-Related Factors

Out of 383 women who underwent cesarean delivery, 322 (84.1%) were urgent. Regarding ASA status, 332 (86.7%) were ASA II, and 51 (13.3%) were ASAIII (Table 2).

**Figure 1:**
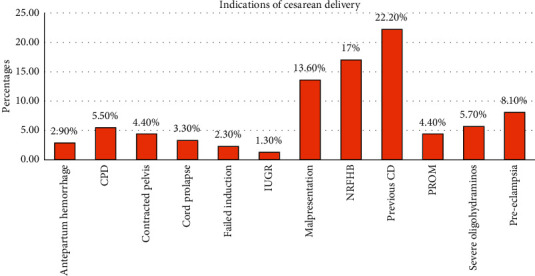
Reasons for cesarean delivery in University of Gondar Comprehensive Specialized Hospital, Northwest Ethiopia, 2019 (*N* = 383). CPD: cephalopelvic disproportion, IUGR: intrauterine growth retardation, NRFHB: non-reassuring fetal heart beat, PROM: premature rapture of the membrane, and CD: cesarean delivery.

Previous cesarean section was the most common indication of cesarean delivery (22.2%) followed by non-reassuring the fetal heart rate pattern (17.0%) (Figure 1).

### 3.3. Intraoperative Anesthesia-Related Factors

Out of the total parturients, 260 (67.9%) were undergoing repeated spinal prick attempts, and 38 (9.9%) of the parturients experienced paraesthesia during needle insertion. Regarding spinal anesthesia-related intraoperative complications, hypotension (60.6%), spinal needle prick pain (49.9%), nausea (46%), nausea and vomiting (16.7%), and shivering (41.1%) were the most happened events, respectively (Table 3).

### 3.4. Postoperative Anesthesia-Related Factors

Among 383 parturients who underwent cesarean delivery, 357 (93.2%), 369 (96.3%), and 346 (90.3%) were free from nausea, nausea and vomiting, and lower back pain, respectively. Out of the total, 125 (32.6%) parturients manifested PDPH, and 94 (24.5%) had pain at the surgical site within two hours immediately postoperative period (Table 4).

### 3.5. Determinants of Maternal Satisfaction after Spinal Anesthesia

The major reasons to refuse SA for the same surgical procedures again in the future were surgical pain, afraid of being awake during the procedure, side effects, and unknown reasons, 27 (5.2%), 13 (3.3%), 11 (2.9%), and 6 (1.6%), respectively. Spinal prick attempts, failed block, PDPH, and antiemetic prophylaxis were factors associated with maternal satisfaction after spinal anesthesia in multivariable logistic regression.

The odds of a parturient with single spinal prick attempts (AOR = 2.08, 95% CI = 1.05–4.11, *p* value = 0.035) were 2 times more likely satisfied than those with multiple attempts. The odds of parturients who have successful spinal block (AOR = 7.17, 95% CI = 3.33–15.43, *p* value < 0.001) were 7 times more likely to have satisfaction than those who got a failed block. The odds of less incidence in postdural puncture headache (AOR = 2.36, 95% CI = 1.33–4.20, *p* value = 0.009) were 2 times more likely to have satisfied than their counterparts. The chance of taking prophylactic antiemetics (AOR = 0.35, 95% CI = 0.19–0.66, *p* value = 0.001) decreased the level of satisfaction by 65% than women who were not taking antiemetic prophylaxis (Table 5).

## 4. Discussion

In the current study, a total of 383 CD parturients were enrolled with overall maternal satisfaction and willingness to choose spinal anesthesia again in the same future surgeries which were 82.3% (95% CI = 78.3–85.9%) and 78.6% (95% CI = 81.5–88.5%), respectively. Our result was similar to a study done by Sadaghi M and his colleagues regarding maternal satisfaction of spinal anesthesia for elective cesarean section which showed that 83.8% of parturients were satisfied. Additionally, 78.5% parturients showed willingness to choose spinal anesthesia in the future surgeries [4]. Another study done by Rashad Siddiqi and Syed Asadullah revealed that the overall level of satisfaction among the parturients who underwent cesarean delivery under spinal anesthesia was 81.4% and 53.66% who would opt for spinal anesthesia in the future [10]. Furthermore, a study done by Morris Senghor and Everlyne Nyanchera regarding determinants of maternal satisfaction with spinal anesthesia for cesarian delivery showed that the overall satisfaction was 85% [23]. However, there are studies that showed levels of maternal satisfaction were higher than this study. A study done by Dharmalingam and Zainuddin in 2013 on maternal satisfaction following SA showed that overall satisfaction and willingness to take it in the future for similar procedures were 97% and 88.5%, respectively [17]. The variation might be explained by use of the 2-point Likert scale (satisfaction or dissatisfaction). Another randomized control trial also reported that the overall parturient satisfaction was 89.48 ± 9.31% with no statistical significance between spinal and epidural anesthesia [24]. The discrepancy might be explained with control of confounding factors and intervention for side effects in their study.

In this study, satisfaction with preanesthesia information about the procedure was 29.1%, which is relatively low compared with Dharmalingam and Ahmad Zainuddin (98%), Shisanya and Morema (36%), and Makoko et al. (67.1%) [13, 17, 23]. This low client satisfaction might be explained with the majority of CD done as emergency (84.1%) and they might be in labor pain that parturients could not concentrate on the preoperative explanation, which is also supported by Shisanya et al. who showed that labour pain has negative impact on satisfaction of parturients with preanesthesia information [23].

Among the demographic variables, educational status is one of the mentioned predictors of maternal satisfaction. A study done by Muneer et al. suggested that maternal satisfaction after spinal anesthesia is negatively associated with higher educational status [14]. This association has been explained by highly educated people have a tendency to extrovert their feelings, information-seeking behavior, and awareness of possible complications.

Studies suggested spinal prick attempt was an independent predictor for maternal satisfaction [2, 25, 26] which is similar to the current finding (AOR = 2.08, 95% CI = 1.05–4.11, *p* value = 0.035). In contrast to this, studies had shown that spinal prick attempts were not significantly associated with satisfaction [17, 18]. These discrepancies could be related due to the use of the 25 G Quincke needle only; however, in this study, we use the 21–25G Quincke spinal needle. Additionally, in the current study, the study area is an institutional hospital; hence, students and junior anesthetists with less skills and experiences may perform multiple attempts that may cause multiple spinal prick; furthermore, we use the 21–25 G spinal needle; the large spinal needle may associate with pin prink.

Studies which were conducted to determine patients' satisfaction after spinal anesthesia concluded that failed block (AOR = 2.28, 95% CI = 0.09–0.87) was the predictor of maternal satisfaction [17, 18], which is similar to the current study (AOR = 7.17, 95% CI = 3.33–15.43, *p* value < 0.001). Multiple studies suggested needle prick pain is an independent predictor for maternal satisfaction after SA [13, 18, 25]. In the current study, even if needle prick pain had a higher frequency (191 (49.9%)), it is not significantly associated with maternal satisfaction. Our finding was parallel with the other study which showed that needle prick pain was not significantly associated with the level of maternal satisfaction [4].

Previous studies showed that postdural puncture headache was the predictor for maternal satisfaction following SA, and less incidence of PDPH was associated with higher level of satisfaction [2, 4, 17, 18], which is similar to the current study (AOR = 2.36, 95% CI = 1.33–4.20, *p* value = 0.009); in contrast, a study done in Iran showed that PDPH was not associated with parturients' satisfaction [4]. Different findings might be explained by preoperative information retention regarding the risk-benefit of SA, and their study participants were only elective parturients. Additionally, postdural puncture headache remains a problem in client satisfaction after spinal anesthesia. The current study showed that the absence of PDPH was two times more likely to have satisfaction with 95% CI = 1.33–4.20, *p* value = 0.003, which was similar to a study done by Siddiqi and his colleagues [17]. The reason for PDPH association with satisfaction might be explained by low preanesthesia information delivery about the possible side effects of the procedure. Another study conducted by Sindhvananda et al. revealed that postdural puncture headache, pruritus, and PONV were predictors of satisfaction [24]. Different findings regarding pruritus and PONV might be explained due to their usage of intrathecal morphine.

Studies documented that postoperative nausea and vomiting was an independent risk factor to determine satisfaction [2, 17, 24, 27], which is in contrast to the current study; the reasons might be in the current study, out of total, 162 (42.3%) parturients were given prophylactic antiemetics and due to less use of intrathecal opioids.

A study done by Ida et al. on factors associated with anesthetic satisfaction after cesarean delivery under neuraxial anesthesia showed that use of intraoperative antiemetics (AOR = 0.71; 95% CI = 0.53–0.94) was positively associated with patient satisfaction [18], which is similar to the current study (AOR = 0.35, 95% CI = 0.19–0.66, *p* value = 0.001).

### 4.1. Limitation of the Study

This study was conducted in a single center, and there was inconsistent adequate supply of medical equipment in the study area which could not truly show the magnitude of the maternal satisfaction level. Additionally, this study did not show a causal relationship between dependent and explanatory variables, as well as using different sizes of the spinal needle can create bias.

## 5. Conclusion

The overall maternal satisfaction receiving spinal anesthesia was low as compared with Leiden perioperative care patient satisfaction. Single spinal prink attempts, successful spinal block, and less incidence of postdural puncture headache can increase maternal satisfaction following spinal anesthesia. Therefore, effective perioperative management and skillful techniques may increase the quality of service and maternal satisfaction.

## Figures and Tables

**Table 1:** Sociodemographic characteristics of parturients at University of Gondar Comprehensive Specialized Hospital, Northwest Ethiopia, 2019 (*N* = 383).

Characteristics		Frequency (*n*)	Percentage (%)	Level of maternal satisfaction	
				Satisfied, *n* (%)	Not satisfied, *n* (%)
Age (year)	<28	227	59.3	180 (47)	47 (12.2)
	≥28	156	40.7	135 (35.3)	21 (5.5)

BMI (kg/m^2^)	≥30	29	7.6	27 (7.04)	2 (0.52)
	<30	354	92.4	288 (75.2)	66 (17.23)

Level of education	No formal learning	99	25.8	88 (23)	11 (2.87)
	Elementary	33	8.6	27 (7)	6 (1.6)
	Secondary	102	26.7	93 (24.28)	9 (2.34)
	Diploma and above	149	38.9	107 (27.93)	42 (10.96)

Marital status	Married	378	98.7	310 (81)	68 (17.7)
	Not married	5	1.3	5 (1.3)	0

BMI (kg/m^2^) = body mass index, kilogram per meter square.

**Table 2:** Maternal and newborn-related factors of parturients at University of Gondar Comprehensive Specialized Hospital, Northwest Ethiopia, 2019 (*N* = 383).

Variables		Frequency (*n*)	Percentage (%)	Level of maternal satisfaction	
				Satisfied, *n* (%)	Not satisfied, *n* (%)
APGAR score at 5 min	10	279	72.8	229 (59.8)	50 (13)
	<10	104	27.2	86 (22.5)	18 (4.7)

Comorbidity	Yes	51	13.3	44 (11.5)	7 (1.8)
	No	332	86.7	271 (70.7)	61 (16)

Pregnancy	Wanted	367	95.7	315 (82.2)	52 (13.5)
	Unwanted	16	4.3	13 (3.4)	3 (0.9)

Previous anesthesia exposure	Yes	91	23.8	82 (21.4)	9 (2.4)
	No	292	76.2	233 (60.8)	59 (15.4)

APGAR: Appearance, Pulse, Grimace, Activity, and Respiration.

**Table 3:** Intraoperative anesthesia-related factors of parturients at University of Gondar Comprehensive Specialized Hospital, Northwest Ethiopia, 2019 (*N* = 383).

Variables		Frequency (*n*)	Percentage (%)	Level of satisfaction	
				Satisfied, *n* (%)	Not satisfied, *n* (%)
Bradycardia	Yes	33	8.6	27 (7.1)	6 (1.6)
	No	350	91.4	288 (75.2)	62 (16.1)

Intraoperative headache	Yes	53	13.8	42 (10.9)	11 (2.9)
	No	330	86.2	273 (71.3)	57 (14.9)

Hypotension	Yes	232	60.6	190 (49.6)	42 (11)
	No	151	39.4	126 (32.9)	25 (6.5)

Light headiness	Yes	158	42.3	128 (33.4)	30 (7.8)
	No	225	57.7	187 (48.8)	38 (10)

Nausea	Yes	176	46	133 (34.8)	43 (11.2)
	No	207	54	182 (47.5)	25 (6.5)

Nausea and vomiting	Yes	64	16.7	45 (11.7)	19 (5)
	No	319	83.3	270 (70.5)	49 (12.8)

Analgesics	Yes	44	11.5	35 (9.1)	9 (2.4)
	No	339	88.5	280 (73.1)	59 (15.4)

Surgical duration (minutes)	≥60	80	20.8	67 (17.5)	13 (3.4)
	<60	303	79.2	248 (64.7)	55 (14.4)

Surgical pain	Yes	33	8.6	17 (4.4)	16 (4.2)
	No	350	91.4	298 (77.8)	52 (13.6)

**Table 4:** Postoperative anesthesia-related factors of parturients at University of Gondar Comprehensive Specialized Hospital, Northwest Ethiopia, 2019 (*N* = 383).

Variables		Frequency (*n*)	Percentage (%)	Satisfaction	
				Satisfied, *n* (%)	Not satisfied, *n* (%)
Nausea and vomiting	Yes	14	3.7	11 (2.9)	3 (0.8)
	No	369	96.3	304 (79.3)	65 (17)

Lower back pain	Yes	37	9.7	29 (7.6)	8 (2.1)
	No	346	90.3	315 (82.2)	31 (8.1)

Shivering	Yes	83	21.7	69 (18)	14 (3.7)
	No	300	78.3	246 (64.2)	54 (14.1)

Postop pain	Yes	94	24.5	79 (20.6)	15 (3.9)
	No	289	75.5	236 (61.6)	53 (13.9)

**Table 5:** Multivariable binary logistic regression on possible risk factors of maternal satisfaction among parturients who underwent spinal anesthesia at University of Gondar Comprehensive Specialized Hospital, Northwest Ethiopia, 2019 (*N* = 383).

Variables		Satisfaction		Odds ratio	
		Satisfied, *n* (%)	Not satisfied, *n* (%)	COR (95% CI)	AOR (95% CI)
Antiemetics given	Yes	147 (90.7)	15 (9.3)	0.32 (0.18–0.60)	0.35 (0.19–0.66)^*∗∗∗*^
	No	168 (76)	53 (24)	1.00	1.00

Block height ≤ T4	Yes	301 (83.8)	58 (16.2)	1.00	1.00
	No	14 (58.3)	10 (41.7)	1.71 (1.60–8.75)	2.11 (0.72–6.25)

Failed block	Yes	14 (45.2)	17 (54.8)	1.00	1.00
	No	301 (80.5)	51 (14.5)	7.17 (3.33–15.43)	7.17 (3.33–15.43) ^*∗∗∗*^

Intraop shivering	Yes	170 (79.4)	44 (20.6)	1.00	1.00
	No	145 (85.8)	24 (14.2)	1.56 (0.91–2.70)	1.57 (0.82–3.01)

Intraop NV	Yes	64	16.4	1.00	1.00
	No	319	83.3	2.3 (1.26–4.31)	2.41 (1.28–4.54)

Needle prick pain	Yes	147 (77)	44 (23)	1.00	1.00
	No	168 (87.5)	24 (12.5)	2.10 (1.22–3.61)	1.72 (0.94, 3.14)

Parity	Primi	136 (97.1)	36 (20.9)	1.00	1.00
	Multi	179 (84.8)	32 (15.2)	1.48 (0.88–2.51)	1.33 (0.71, 2.49)

Paraesthesia	Yes	27 (71.1)	11 (28.9)	1.00	1.00
	No	288 (83.5)	57 (16.5)	2.06(0.97–4.39)	1.24 (.49–3.14)

Spinal prick attempts	1	107 (87)	16 (13)	1.67 (0.91–3.07)	2.08 (1.05–4.11) ^*∗∗∗*^
	≥2	208 (80)	52 (20)	1.00	1.00

Urgency	Elective	55 (90.2)	6 (9.8)	2.19 (0.90–5.31)	2 (0.70–5.66)
	Emergency	260 (80.7)	62 (19.3)	1.00	1.00

Postdural puncture headache	Yes	93 (74.4)	32 (25.6)	1.00	1.00
	No	222 (86)	36 (14)	2.12 (1.24–3.62)	2.36 (1.33–4.20) ^*∗∗∗*^

^*∗∗∗*^Significant at multivariable logistic regression with *p* value <0.05. T4: thoracic level four; NV: nausea and vomiting. 1.00 = reference.

## Data Availability

The datasets used and/or analyzed during the current study are available from the corresponding author upon reasonable request.

## Abbreviations

APGAR: Appearance, Pulse, Grimace, Activity, and Respiration
ASA: American Society of Anesthesiologists
BMI: Body mass index
CD: Cesarean delivery
CI: Confidence interval
CPD: Cephalopelvic disproportion
COR: Crude odds ratio
IUGR: Intrauterine growth restriction
IQR: Interquartile range
LPPSQ: Leiden perioperative care patient satisfaction questionnaire
MSc: Master of Science
OR: Odds ratio
PDPH: Postdural puncture headache
PONV: Postoperative nausea and vomiting
PROM: Premature rupture of the membrane
SA: Spinal anesthesia
SPSS: Statistical Package for Social Studies
WHO: World Health Organization.
